# Engineering exosomes in the treatment of sensorineural hearing loss: from basic research to clinical applications

**DOI:** 10.3389/fnmol.2026.1886218

**Published:** 2026-08-21

**Authors:** Siqi Wang, Juanjuan Li, Bingxing Yang, Mengdi Li, Man Yu, Peng Zhang, Suwen Bai, Xianhai Zeng

**Affiliations:** 1 Department of Graduate and Scientific Research, Zunyi Medical University Zhuhai Campus, Zhuhai, Guangdong, China; 2 Department of Otolaryngology, Longgang Otolaryngology Hospital and Shenzhen Key Laboratory of Otolaryngology, Shenzhen Institute of Otolaryngology, Shenzhen, Guangdong, China

**Keywords:** engineered exosomes, sensorineural hearing loss, blood-labyrinth barrier, targeted drug delivery, cochlear microenvironment

## Abstract

Engineered exosomes demonstrate good biocompatibility, barrier-crossing ability, and programmable drug-loading capacity. Recently, they have steadily emerged as a promising area of study for precision intervention in sensorineural hearing loss. This review focuses on numerous main topics, including the selection of exosome sources and donor-cell pretreatment techniques, surface targeting strategies, therapeutic cargo loading methods, the mechanisms by which exosomes cross the blood-labyrinth barrier, and recent advancements in research on the role of exosomes in hair cell protection, spiral ganglion neuron protection, supporting-cell plasticity, and cochlear microenvironment remodeling. This review also examines the key challenges in the clinical translation of exosomes. It comprehensively reviews direct cochlear exosome evidence for hair cell protection and auditory protection, separating these results from indirect non-cochlear exosome evidence and theoretical engineering approaches for spiral ganglion neuron protection, supporting-cell-mediated regeneration-related mechanisms, and cochlear neurovascular microenvironment remodeling. In addition to outlining a framework that combines targeted delivery and functional regulation for sensorineural hearing loss, this review highlights the hearing-protective potential of engineered exosomes in preclinical models and requires further experimental validation. Despite the positive outlook, there are still notable issues with the durability of therapeutic effects, mechanistic clarity, and clinical translatability. Future studies should prioritize standardization of extracellular vesicle reporting, dose reproducibility, clinically relevant models, and long-term functional outcomes.

## Graphical Abstract

**Figure:**
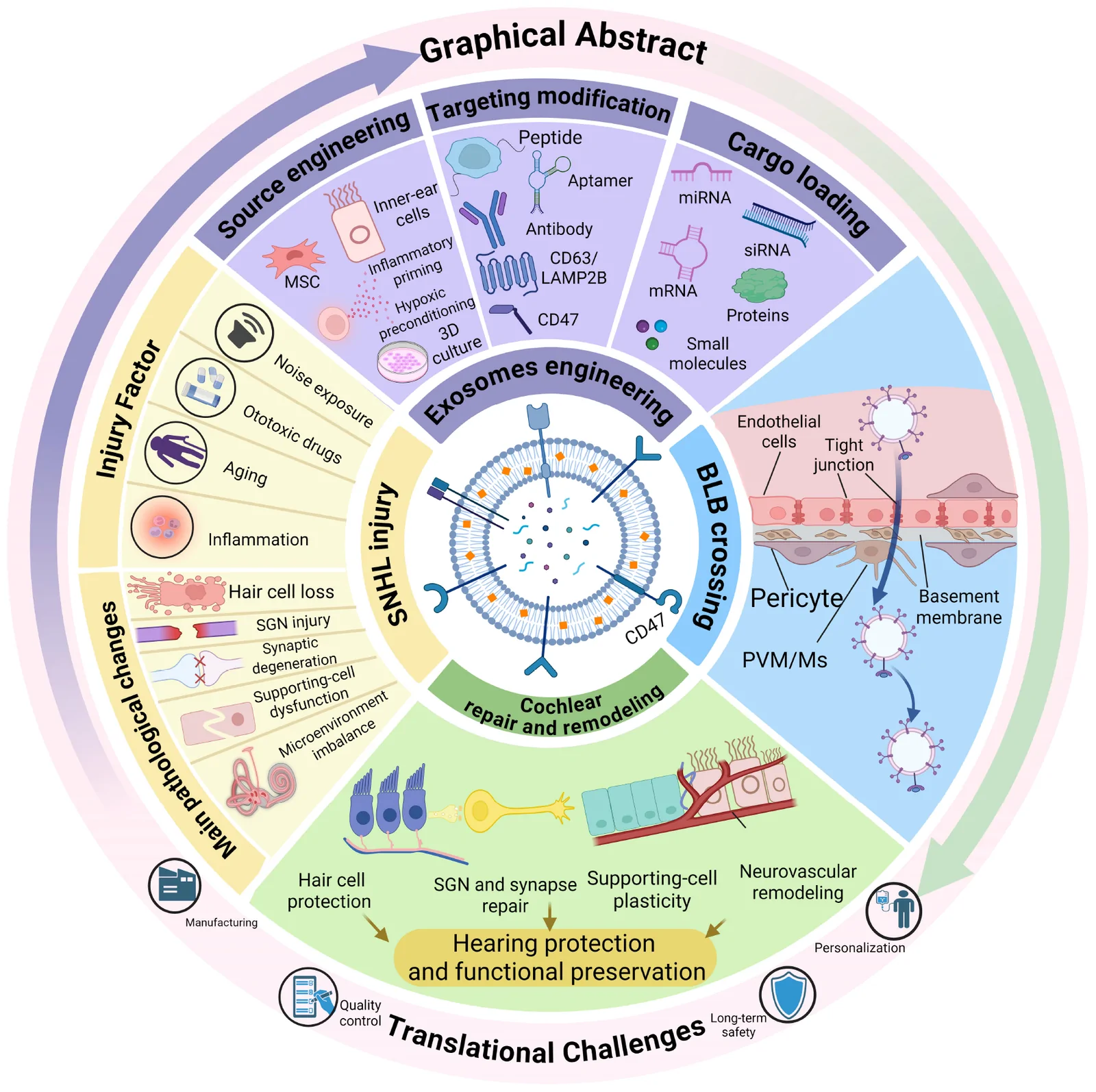
Overview of engineered exosome strategies for sensorineural hearing loss, including source engineering, surface targeting, cargo loading, blood-labyrinth barrier (BLB)-oriented delivery, cochlear protection and remodeling, and translational considerations. The figure was created with BioRender.com.

## 1 Introduction

Sensorineural hearing loss (SNHL) is a common type of hearing impairment primarily involving damage to hair cells, spiral ganglion neurons, and cochlear-related structures that affects sound perception, conversion, and neural transmission. The most recent epidemiological data demonstrate that the aging population, increased noise exposure, and persistent ototoxic factors are all contributing to the global burden of hearing loss, making SNHL an increasingly prominent public health concern (GBD 2019 Hearing Loss Collaborators, 2021). Currently, clinical interventions primarily rely on hearing aids and cochlear implants to achieve auditory rehabilitation. However, since these techniques primarily focus on enhancing sound transmission and auditory compensation, they are unlikely to reverse cochlear tissue damage or stop the pathological progression of the disease (Orji et al., 2020; Chadha et al., 2021). As a result, developing new approaches to treat SNHL is required.

Currently, SNHL treatment is gradually shifting from basic functional compensation toward precise therapies that target damaged cells, neural connections, and the cochlear microenvironment. The blood-labyrinth barrier (BLB) has long been acting as an obstacle to the effectiveness of pharmacological treatment in achieving long-term therapeutic results in the inner ear. Additionally, the steady-state maintenance role of the BLB considerably prevents medicines from entering the cochlear tissue following systemic delivery, which leaves local regions with inadequate drug exposure (Zhang et al., 2012; Neng et al., 2013). Similar challenges will also occur in the event of local administration, such as short residence time of the drug in the cochlea, the inconsistent efficiency of transmembrane transport, and the uneven distribution along the cochlear axis (Lukashkin et al., 2020).

Exosomes have become increasingly important carriers for inner-ear drug delivery due to their superior biocompatibility, minimal immunogenicity, and capacity for biological barrier penetration (Ostrowski et al., 2010). Exosomes have the potential to improve cross-barrier transport efficiency, support local enrichment in the cochlea, and produce cell-specific intervention effects through engineered techniques such as surface modification, therapeutic cargo delivery, and source optimization. Existing research indicates that exosomes exhibit protective and reparative potential in models of cisplatin-induced ototoxicity and noise-induced hearing loss (Tsai et al., 2021; Min et al., 2023). This comprehensive narrative review will cover the main strategies of exosome engineering, the mechanisms underlying exosome transport across the BLB, and the potential of exosomes to modify the cochlear microenvironment, aid in cochlear repair, and create a favorable microenvironment for responses related to regeneration. Three types of evidence will be used to categorize the studies covered in this review: direct cochlear exosome evidence, indirect exosome evidence from non-cochlear systems, and theoretical engineering techniques.

Currently, clinical and mechanistic research is the primary focus of this field. This review summarizes the research findings, assesses the strength and limitations of the evidence, and investigates the exosome source, isolation and characterization techniques, administration routes and dosing schedules, dose units, animal age and sex, sample size, follow-up duration, ABR/DPOAE outcomes, and reproducibility across models. Moreover, it addresses the important challenges that need to be resolved for clinical translation.

We searched PubMed/MEDLINE, Web of Science Core Collection, and Scopus for English-language literature published between January 2010 and June 2026. The main search terms included “exosome,” “extracellular vesicle,” “engineered exosome,” “sensorineural hearing loss,” “cochlea,” “blood-labyrinth barrier,” “hair cell,” “spiral ganglion neuron,” and “drug delivery.” Original studies and reviews related to exosome engineering, cochlear delivery, auditory protection or repair, and clinical translation were included, with priority given to studies offering direct cochlear evidence. Duplicate publications and studies clearly unrelated to the subject were excluded.

## 2 Core strategies and technical approaches in exosome engineering

### 2.1 Selection and pretreatment of exosome sources

The engineering design process begins with the selection of the exosome source. The protein cargo composition and functional profile of exosomes can change depending on the lineage background and differentiation status of donor cells. The selection of the source is a crucial first step that affects the stability, biological behavior, and potential for transmission (La Greca et al., 2018). The baseline biological properties of the vesicles and their suitability for scalable, xeno-free production with sufficient batch-to-batch consistency are determined by the donor-cell system from the perspective of translational application (de Almeida Fuzeta et al., 2020).

At present, mesenchymal stem cells (MSCs) are the most commonly used cellular platform for exosome production. Their widespread utilization reflects the well-established expandability of MSCs, relatively high vesicle secretion, and vast translational experience (Haraszti et al., 2018). MSCs of the same type can secrete exosomes with notably varied cargo profiles under diverse differentiation or culture conditions. Source engineering entails the selection and modification of donor cells to predetermine the natural biological characteristics of exosomes (Wang et al., 2018). Studies have demonstrated that dynamic 3D culture can further boost the output of exosomes from human bone marrow-derived MSCs and modify their particle size distribution and functional components, indicating that the culture scaffold is a crucial feature of source engineering (Yuan et al., 2022).

The functional programming of exosomes is influenced by various pretreatment techniques in addition to culture conditions. The functional reprogramming of exosomes really starts during the vesicle formation stage, which is evident by the ability of hypoxic pretreatment to enrich MSC-derived exosomes with miRNAs linked to cell survival and tissue repair (Zhu et al., 2018). Inflammatory preactivation, like hypoxic pretreatment, can improve the immunomodulatory potential of MSC-derived exosomes and encourage macrophage polarization toward an anti-inflammatory phenotype (Domenis et al., 2018). Exosomes derived from umbilical cord MSCs have been indicated to lessen cisplatin-induced outer hair cell loss and increase cochlear tissue repair (Tsai et al., 2021). Additionally, exosomes derived from heat shock-treated bone marrow MSCs can alleviate cisplatin-induced ototoxicity by preventing the activation of the NLRP3 inflammasome in the cochlea through HSP70 enrichment (Yang et al., 2022b). Accordingly, exosomes can be a viable strategy to combat drug-induced deafness and general hearing loss.

Exosomes derived from various cell sources exhibit different characteristics related to their origins, representative models, cochlear targets, and forms of evidence (Table 1; Breglio et al., 2020; Lai et al., 2020; Warnecke et al., 2020; Yang et al., 2021, 2022a; Hao et al., 2022). Careful comparison of exosomes from distinct sources is required since many studies use diverse donor cell sources, culture conditions, pretreatment plans, isolation techniques, exosome purity, and preservation conditions–all of which may change exosomal cargo and biological activity. Direct comparisons between exosomes derived from MSCs, inner-ear, and progenitor cells are limited since certain inner-ear studies have not sufficiently reported sample size, animal age, sex, or batch-to-batch reproducibility. Therefore, source selection and pretreatment together influence the inherent properties of the exosomes, their suitability for further engineering, and the potential therapeutic effect. They also comprise an ongoing design process inside the same engineering module.

**TABLE 1:** Biological characteristics and applications of exosomes from different sources in sensorineural hearing loss-related research.

Exosome source	Source characteristics	Representative model	Primary target cells	Key features	Main applications	Evidence level	Representative literature
Inner ear supporting cells	It exhibits a high degree of tissue specificity and is more closely linked to the endogenous paracrine system of the cochlea	Models of hair cell damage	Hair cells	Carries protective molecules and participates in the endogenous stress defense mechanisms of cochlea	Endogenous hair cell protection	*In vitro* and *in vivo* studies	Breglio et al., 2020
Inner ear stem cells	More closely related to the protection and regeneration of sensory epithelium	*In vitro* model of gentamicin ototoxicity	Hair cells	Regulation of oxidative stress and apoptosis-related pathways	Hair cell protection and regeneration-related effects	*In vitro* studies	Lai et al., 2020
Spiral ganglion progenitor cells	More closely aligned with cochlear nerve damage and the local repair environment	Model of ischemia-reperfusion-induced hearing loss	Cochlear neural structures and the local inflammatory environment	Inhibits the expression of inflammatory factors and reduces cell apoptosis	Local anti-inflammatory protection	*In vivo* animal studies	Yang et al., 2021
Neural progenitor cells	Better suited for delivering neuromodulatory drugs	Model of ischemia-reperfusion-induced hearing loss	Inflammatory microenvironment	Enriching functional nucleic acids can enhance anti-inflammatory effects	Engineered nucleic acid delivery	*In vitro* and *in vivo* animal studies	Hao et al., 2022
Umbilical cord MSCs	The supply is relatively stable, the growth potential is strong, and the prospects for commercialization are promising.	Cisplatin ototoxicity model	Outer hair cells and damaged cochlear tissue	Attenuates outer hair cell loss and improves the condition of damaged tissue	Protection of hair cells and cochlear repair	*In vitro* and *in vivo* animal studies	Tsai et al., 2021
Bone marrow MSCs	The research foundation is relatively well-established, and there has been extensive research on pretreatment processes.	Cisplatin ototoxicity model	Hair cells and the immune microenvironment	Antioxidant, anti-apoptotic, and anti-inflammatory	Therapies for hair cell protection and engineered enhancement	*In vitro* and *in vivo* animal studies	Yang et al., 2022a
Human MSCs	Possesses strong paracrine repair potential	Model of noise-induced hearing loss	Structures associated with hair cells	Activate pathways related to post-injury protection and repair	Protection against noise-induced hearing loss	*In vivo* animal studies	Warnecke et al., 2020
Small extracellular vesicles enriched with neurotrophic factors	A greater emphasis on functional load design can enhance neuroprotective effects	Model of noise-induced hearing loss	Spiral ganglion neurons and synaptic units	Delivers neurotrophic factors to promote neuroprotection	Neuronal protection and synaptic repair	*In vivo* animal studies	Min et al., 2023

The biological characteristics and therapeutic effects of exosomes vary depending on their source; research findings should be interpreted in conjunction with the source cells, preparation methods, and engineering strategies.

### 2.2 Engineering exosomes for targeted modification

**FIGURE 1:**
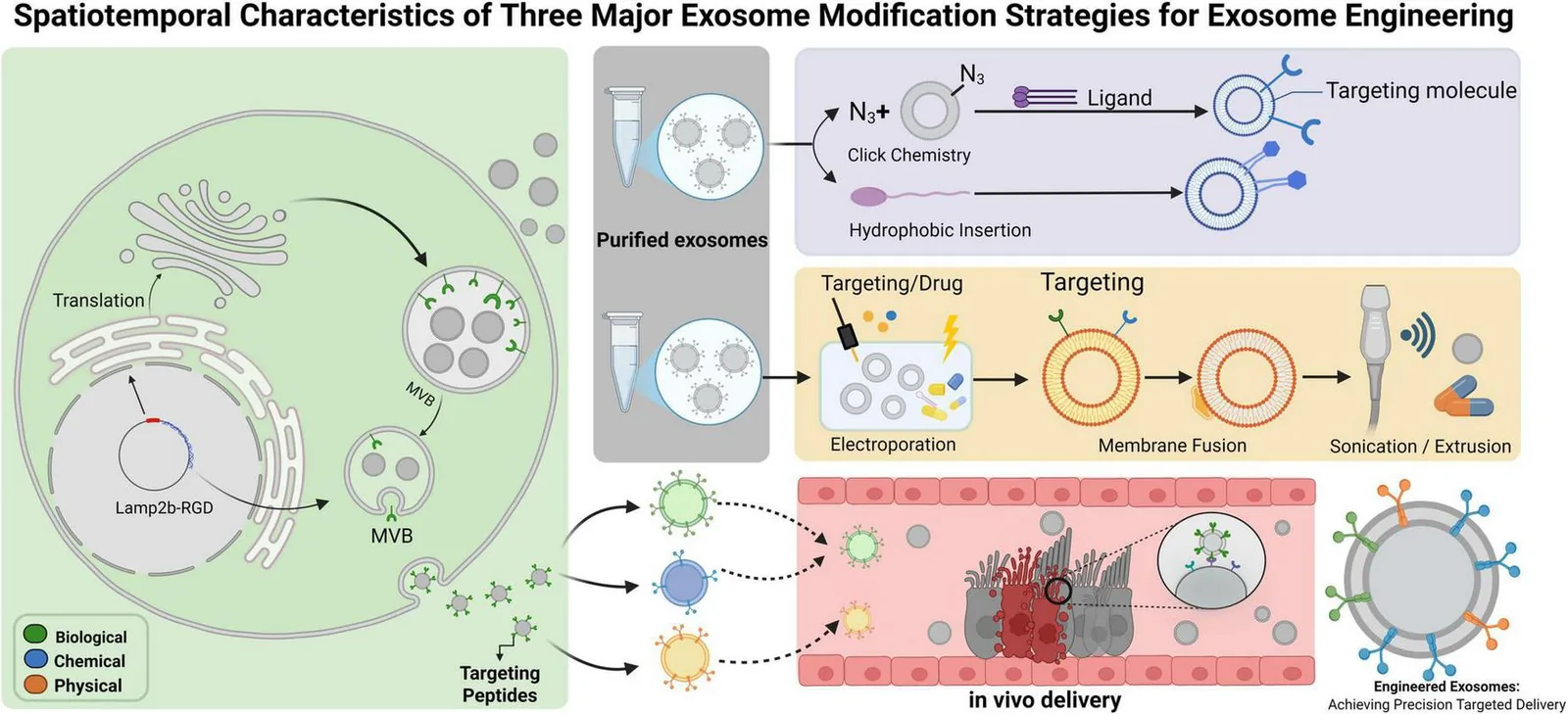
Spatiotemporal characteristics of three major exosome modification strategies for exosome engineering. Three major modification strategies for engineered exosomes are: biological modification, chemical modification, and physical modification. Biological modification is primarily completed within donor cells, allowing the display of targeting peptides or membrane proteins on the exosome surface through genetic engineering; chemical modification is mostly done following exosome isolation and can connect targeting molecules through click chemistry, hydrophobic insertion, or covalent conjugation; physical modification loads drugs or functional molecules via electroporation, membrane fusion, ultrasound, or extrusion. The three strategies differ in operation stages and modification methods; however, they all aim to improve the targeted delivery capacity of exosomes. Green, blue, and orange represent biological, chemical, and physical modifications, respectively. The figure was created with BioRender.com.

Natural exosomes inherently exhibit a certain degree of tissue tropism; however, this natural membrane phenotype does not yet meet the requirements for precise delivery, and surface modification is needed to enhance their ability to recognize target tissues and cells (Wiklander et al., 2024). According to the stage of modification intervention, exosome engineering can be divided into biological modification during vesicle biogenesis, as well as chemical and physical modification after exosome separation (Figure 1). The recognition interface of natural exosomes can be engineered through biological, chemical, and physical modification strategies. Biological modifications enable cells to secrete targeting peptides or fusion proteins via membrane proteins such as LAMP2B and CD63, while chemical and physical changes offer complementary post-isolation modifications for pure exosomes (Gao et al., 2018; Piffoux et al., 2018; Zou et al., 2019). These modification techniques are intended to promote steady circulation, barrier-crossing transport, tissue accumulation, and target cell uptake during exosome delivery *in vivo*.

Current engineering approaches integrate several functions to overcome these problems. Exosomes are transformed from natural vesicles to programmable delivery vehicles by methods such as RVG-Lamp2b-modified exosomes and the EXOtic platform, which combine surface targeting, cargo loading, and intracellular release into a single engineering system (Liu et al., 2015; Kojima et al., 2018; Min et al., 2023). Targeting ligands can be modularly added to the exosome surface through chemical alteration following separation without affecting the donor cells. The targeting peptides or cargo molecules are linked to exosomes by the anchoring peptide CP05 to the exosomal membrane protein CD63 (Gao et al., 2018). Diacyl-lipid-conjugated aptamers are added to the lipid bilayer of exosomes to improve ligand-dependent cellular absorption and drug delivery (Zou et al., 2019).

Physical modification adds functional components to exosomes by momentarily disrupting or rebuilding the vesicle membrane. When exosomes and functionalized liposomes fuse, hybrid vesicles with exogenous membrane and soluble components are formed, retaining part of the intrinsic contents of the original vesicles (Piffoux et al., 2018). Electroporation, ultrasound, freeze-thaw cycles, and extrusion can all encourage cargo loading or membrane structure remodeling. The primary quality control concerns of these techniques include modification in vesicle size, membrane integrity, surface protein topology, aggregation state, and biological activity.

Research in models of cerebral ischemia has demonstrated that post-isolation surface re-engineering can provide a stable and adjustable ligand-presenting interface, leading to improved trans-barrier delivery efficiency and lesion enrichment capacity (Tian et al., 2018; Dooley et al., 2021; Wiklander et al., 2024). These platforms, which offer engineering strategies pertinent to BLB-targeted delivery, are mostly based on models of the central nervous system and blood-brain barrier.

According to recent studies, TSPAN2 and TSPAN3 have more engineering potential than CD63, the canonical tetraspanin. The selection of various membrane scaffold proteins will directly impact the particle size, surface marker expression, and cell absorption efficiency of the engineered exosomes. These results suggest that ligand selection and compatibility with the display scaffold simultaneously determine the targeting performance (Zhang et al., 2022; Zheng et al., 2023). Targeted alteration for SNHL must also consider the unique properties of BLB and cochlear cells, with particular focus on the distribution, cellular uptake, and functional release of the exosomes following their entry into the cochlear tissue. BLB transport and local cochlear-cell recognition should be viewed as sequential parts of a single delivery process for SNHL targeting techniques to be effective. Performance throughout the whole delivery cascade, including barrier crossing, target-cell uptake, and functional cargo release, is critical to the success of targeted modification. The benefits observed *in vitro* can only be converted into practical therapeutic effects *in vivo* in this manner.

### 2.3 Exosome drug delivery engineering

The primary objective of cargo-loading engineering is to load therapeutic drugs effectively while maintaining vesicle membrane integrity, colloidal stability, and cargo activity, and permitting functional release at the target site. The degree of membrane damage and the efficacy of various exogenous drug-loading techniques vary. While loading can be accomplished using a variety of techniques, including freeze-thaw, ultrasonication, and extrusion, there are notable variations in drug-loading capacity, release kinetics, and the extent of structural damage to the exosome (Haney et al., 2015). There are still marked differences in the actual cargo load, the physical characteristics of vesicles, and their final functional performance, despite the fact that different techniques can encourage the loading of nucleic acids into exosomes (Guarro et al., 2025).

Consequently, loading efficiency and the biological functional integrity of the vesicles following loading must be considered when assessing drug loading. A more regulated strategy based on molecular design is gradually replacing the earlier emphasis on empirical co-secretion in the field of drug delivery via exosomes. For instance, incorporating mRNA into small exosomes to produce an adaptive architecture that permits the docking of various targeting ligands to achieve synergistic optimization of cargo loading and tissue targeting (Dong et al., 2023). The efficiency of protein cargo entrance into vesicles can be greatly increased by improving the interactions between proteins and lipid rafts, which also improves subsequent functional delivery in addition to optimizing the loading framework. This indicates that cargo sorting and functional output are two closely related phases of the same engineering process (Peruzzi et al., 2024). The mRNA loading and delivery capacity are further improved by a hybrid platform that combines exosomes and lipid nanoparticles. The issue of inadequate payload capacity in natural vesicles can be effectively resolved by this biomimetic hybrid (Wang et al., 2025).

Neurotrophic factors are important candidate cargoes for inner ear drug delivery systems, as evidenced by the obvious protective effects of small extracellular vesicles prepared from Lentivirus-BDNF infected MSCs in models of noise-induced hearing loss (Koehler et al., 2017). Overexpression of microRNA-21 in exosomes derived from neural progenitor cells reduced inflammation in the cochlea, thereby mitigating hearing damage in a model of ischemia-reperfusion-induced hearing loss (Pan et al., 2025). This suggests that nucleic acid-based therapeutic agents have a transient paracrine protective effect and that engineered exosomes can deliver gene-editing agents in a model of adult-onset hereditary hearing loss. This establishes a foundation for employing engineered exosomes to perform more advanced nucleic acid interventions. Moreover, the engineering potential of exosome-like carriers for nucleic acid transport has also been demonstrated by the efficient delivery of RNA therapies using exosomes derived from red blood cells (Usman et al., 2018).

There is increasing evidence that the development of drug delivery systems for SNHL necessitates a systematic approach that considers the particular underlying causes and the treatment window. Anti-inflammatory, anti-apoptotic, and neurotrophic support are prioritized for acute ototoxic damage and interventions after noise exposure; in contrast, nucleic acid carriers with sustained expression or more effective programmed delivery systems are needed for chronic degenerative or genetic damage. Studies of drug-loaded exosomes should report exosome dose units, loading efficiency, free-drug removal, and release kinetics to identify the source of therapeutic efficacy. It is essential to conduct a comprehensive examination that incorporates cargo selection, loading techniques, targeting modification, and the pathological state of the inner ear to advance engineered exosomes from a proof-of-concept stage into a truly translatable therapeutic platform.

## 3 Mechanism of engineered exosomes for targeted drug delivery penetrating the BLB

### 3.1 Structural characteristics of the BLB and the challenges of drug delivery

Blood-labyrinth barrier, a complex barrier unit, is composed of endothelial cells, pericytes, perivascular resident macrophage-like melanocytes (PVM/Ms), and a common basement membrane. These cells interact continuously and closely to maintain stability (Ostrowski et al., 2010; Lukashkin et al., 2020). BLB can affect the efficiency with which drugs enter the cochlea, their distribution within the cochlea, and local exposure levels during the delivery of drugs to the inner ear. BLB is a dynamically regulated, stress-responsive barrier whose permeability can change in response to injury stimuli. Barrier structures are damaged when this steady state is disrupted, followed by a continuous inflammatory cascade.

**FIGURE 2:**
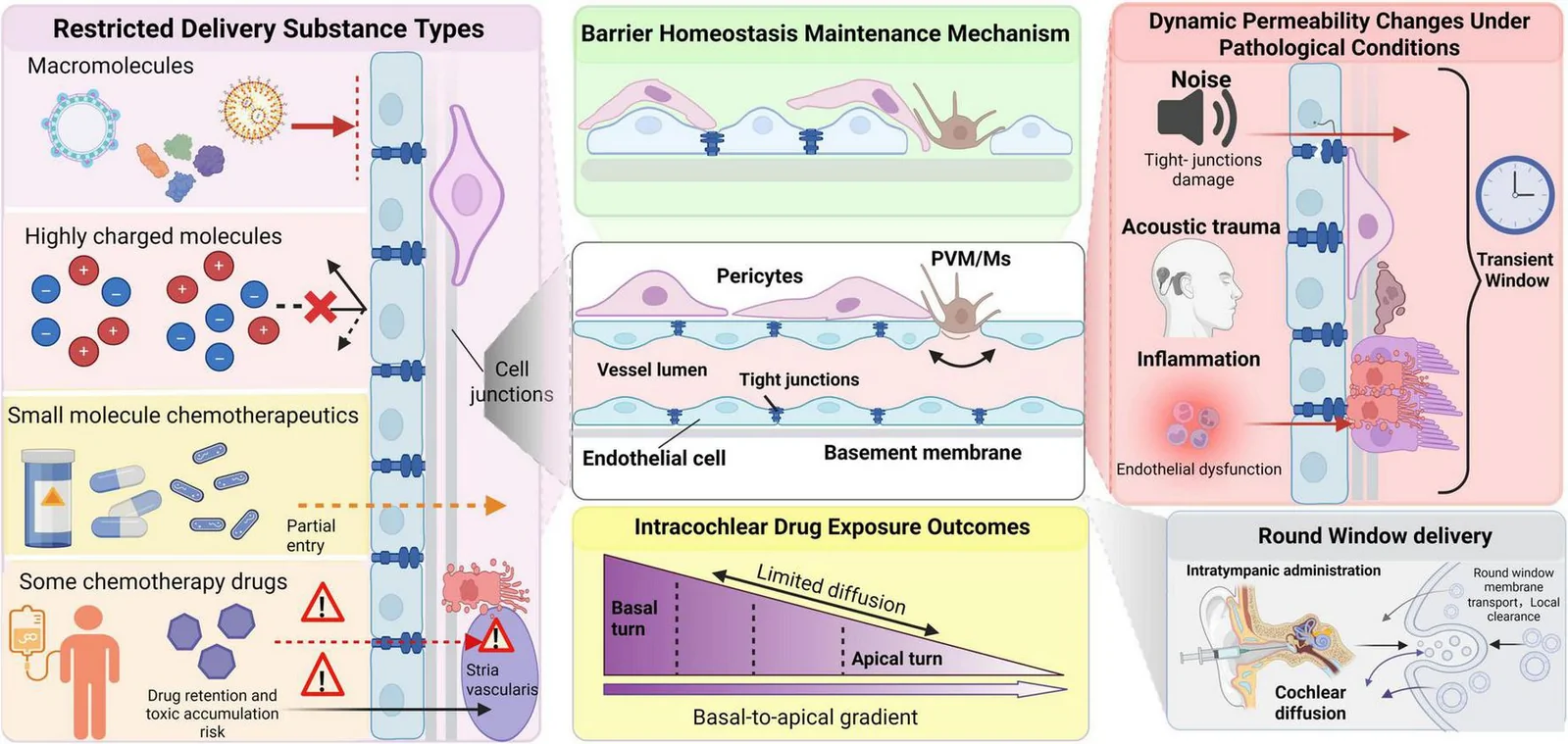
Major challenges in drug delivery for SNHL. There are significant obstacles and local exposure restrictions in drug delivery for sensorineural hearing loss. BLB is maintained by vascular endothelial cells, tight junctions, pericytes, perivascular resident macrophage-like melanocytes, and a shared basement membrane, which prevents the entry of macromolecules, highly charged molecules, and certain drugs into the cochlea. Barrier permeability can be momentarily enhanced by noise exposure, acoustic trauma, and inflammatory conditions; however, it is challenging to regulate the duration of increased permeability and the safety range. Drugs can enter the cochlea through the round window membrane after transtympanic administration, although they frequently generate a concentration gradient that declines from the basal to the apical turn and is influenced by local clearance. Consequently, the crucial challenge in SNHL drug delivery is to achieve adequate, uniform, predictable, and safe local drug exposure while preserving cochlear homeostasis. The figure was created with BioRender.com.

Exposure to noise triggers an aberrant reaction in perivascular macrophages of the cochlea and damages tight-junction structures. Capillary degeneration may result from myofibroblastic transformation of pericytes caused by acoustic trauma. The integrity of the endothelium layer and the status of cellular junctions within the BLB can be directly affected by inflammatory conditions. Sustained toxic accumulation can result from the persistence of cisplatin in the cochlea for extended periods and its accumulation to high levels in the stria vascularis region (Figure 2; Zhang et al., 2013; Breglio et al., 2017; Hou et al., 2020; Sekulic et al., 2023).

Drug distribution within the cochlea remains influenced by diffusion restrictions between various fluid compartments, regional clearance differences, and concentration gradients from the basal to the apical turn after local administration. The problem of uneven drug distribution inside the cochlea cannot be solved by only avoiding or partially penetrating the BLB, which limits the final therapeutic efficacy (Plontke et al., 2007). Barrier permeability is also regulated by molecules involved in ion transport. The permeability of the endothelial cells in the BLB rises when NHE6 is absent, or when NKCC1 is inhibited (Sekulic-Jablanovic et al., 2022). The permeability of BLB also increases within a brief window of time after noise exposure, facilitating more efficient delivery of blood-borne treatments to the inner ear. Although the barrier itself has a dynamic, time-dependent window of opportunity, it is challenging to directly translate this window into a stable, controllable treatment pathway since it is subject to clear time constraints and safety limits. The round window membrane offers an alternative local delivery route.

The round window membrane is not merely a passive diffusion interface. The localized exosome network in the round window membrane may play a role in regulating drug transport in the inner ear, providing evidence that this localized delivery pathway can also be engineered for therapeutic applications (Holdsclaw et al., 2025). The structure of the BLB is not entirely static. The permeability of the paracellular space can rise under certain stimuli within a limited time window, increasing the effectiveness of drug delivery to the inner ear. However, this opening cannot be directly used as a long-term treatment modality because it is obviously time-dependent and has safety restrictions (Wang et al., 2024). Therefore, the fundamental issue in drug delivery for SNHL is to achieve adequate, consistent, and predictable local drug exposure while completely maintaining the internal stability of the cochlea.

### 3.2 Core mechanisms underlying the engineering of exosomes to penetrate the BLB

Strategies for drug delivery across the BLB must be developed based on the molecular targets of the barrier itself; although knowledge of the blood-brain barrier can serve as a reference, it cannot be directly extrapolated. LRP1 as a receptor at the BLB interface and LRP1-binding peptide IETP2 as an inner ear-targeting ligand are supported by direct inner ear data. However, peptide-coupled small molecules are currently used to verify IETP2-mediated transport across the BLB. Consequently, direct BLB evidence for LRP1 and IETP2 suggests that they may be useful for exosome surface modification (Shi et al., 2022). Exosomes can act as carriers for delivery across the BLB because of their lipid bilayer structure, which efficiently encapsulates and protects miRNAs, siRNAs, mRNAs, and protein cargo, while also enabling these cargoes to continue functioning after entering the recipient cell (Valadi et al., 2007; Pegtel et al., 2010). Intravenous injection of BDNF-loaded MSC-derived small exosomes can improve BDNF protein expression in the cochlea and reduce noise-induced hearing loss in the SNHL-specific model, offering direct evidence of the systemic administration of engineered small exosomes to the cochlea (Min et al., 2023). Currently, this study has not clarified the involved BLB receptors, endothelial cell uptake pathways, and transcytosis routes.

**FIGURE 3:**
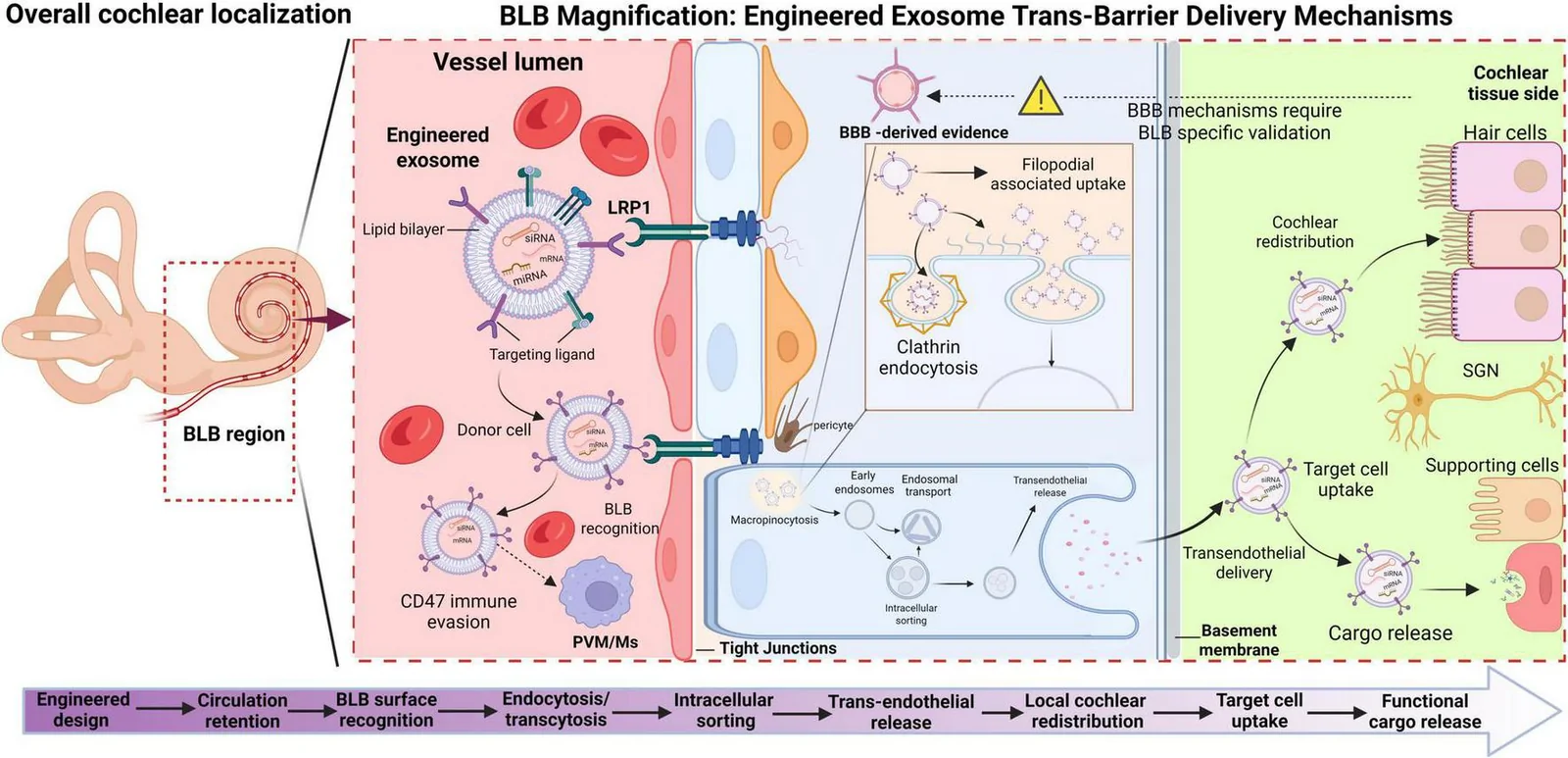
Blood-labyrinth barrier (BLB) magnification: engineered exosome trans-barrier delivery mechanisms. Schematic illustration of engineered exosome-mediated trans-BLB delivery. This schematic also includes direct evidence from the inner ear (including LRP1/IETP2-mediated inner ear targeting and cochlear delivery of BDNF-loaded small exosomes after systemic administration), and the mechanisms of uptake and transcytosis deduced from blood-brain barrier and non-cochlear extracellular vesicle studies must be directly confirmed in the BLB later. The left side depicts the overall positioning of the cochlea and the BLB region; the middle-enlarged image indicates the systemic retention of exosomes within the blood vessel lumen, BLB surface recognition, endocytosis/transcytosis, intracellular sorting, and trans-endothelial release; the right side depicts their local distribution within cochlear tissue, target cell uptake, and functional cargo release. Engineered exosomes enhance the potential of BLB-crossing delivery through surface targeting ligands, LRP1-related recognition, CD47-mediated immune evasion, and nucleic acid or protein cargo loading. BLB, blood-labyrinth barrier; LRP1, low-density lipoprotein receptor-related protein 1. The figure was created with BioRender.com.

Exosomes can enter recipient cells through clathrin-mediated endocytosis, macropinocytosis, and filopodia-associated endocytic pathways after coming into contact with them. They subsequently undergo endosomal transport and intracellular sorting. Although these pathways are supported by extensive research on exosome uptake, their precise functions in the uptake and transcellular transport by BLB endothelial cells in the inner ear have not yet been established. These processes provide the necessary framework for exosomes to achieve barrier-crossing delivery (Figure 3; Tian et al., 2014; Heusermann et al., 2016). The efficiency with which exosomes reach the BLB interface is affected by the source of the donor cells, the administration route, and the composition of surface molecules (Wiklander et al., 2015). Particularly, the expression of integrins and other molecules on the membrane surface affects the tendency of exosomes to interact with resident cells in specific organs. This natural distribution pattern offers an essential basis for subsequent engineering applications (Hoshino et al., 2015). The data for this interaction were mainly provided by the non-cochlear models and can be used as a mechanistic foundation to infer targeted behavior.

Accordingly, the barrier-crossing potential of exosomes results from the combined effects of their natural membrane phenotype, tendency for *in vivo* distribution, and intracellular sorting. The blood-brain barrier and other non-cochlear models mostly support the following surface ligands, endothelial cell sources, and immune evasion strategies, which can be used as indirect evidence for BLB-oriented exosome design. Exosomes can effectively pass through the blood-brain barrier through surface ligand modification and deliver siRNA to brain tissue (Min et al., 2023). Endothelial-derived exosomes can improve the effectiveness of siRNA delivery across the endothelial barrier and its functional output in brain-endothelial-related models. This enhancement in barrier-crossing efficiency can be attained through source-cargo matching (Yang et al., 2017).

CD47 is not a BLB recognition receptor and primarily extends the circulation time of the vesicle system by decreasing the phagocytosis and clearance of vesicles by macrophages. Consequently, increasing the CD47-mediated immune-evasion capacity of exosomes helps decrease macrophage-mediated phagocytosis, extend the retention time of exosomes in the body, and raise the likelihood of contacting target tissues or barrier interfaces (Ben et al., 2022). However, the receptor distribution and microenvironment of the BLB differ slightly from those of the blood-brain barrier. The exosomes binding to endothelial cells is only the start of transendothelial delivery; the final delivery result is influenced by subsequent intracellular transport, endocytosis, and local redistribution within the cochlea (Morad et al., 2019; Zhang et al., 2020).

According to direct inner ear data, the endothelial cells of the BLB are the first barrier-side targets that need to be identified following drug delivery via the exosome system. The downstream targets in the cochlea are hair cells, supporting cells, spiral ganglion neurons, and stria vascularis cells after they have crossed the BLB. There is currently insufficient direct inner ear evidence to demonstrate that LRP1-targeted engineered exosomes can be selectively delivered to various cells that express LRP1.

## 4 Protective, reparative, and regeneration-related potential of engineered exosomes in SNHL

### 4.1 Mechanisms of hair cell protection mediated by engineered exosomes

Exosomes released by endogenous supporting cells in the cochlea mediate the protection of sensory hair cells through a mechanism involving heat shock protein 70, demonstrating that exosomes are also a component of the defense network of the stress of the inner ear (Breglio et al., 2020). The mechanism of action of exosomes at the hair cell level aligns with the inherent protective mechanisms of the cochlea. Engineered exosomes can directly contribute to regulating cell death pathways through their membrane surface and the active molecules they contain, while delivering drugs. According to recent research, exosomes from a variety of sources can successfully reduce hair cell apoptosis, auditory threshold shifts, and alleviate oxidative stress in different injury models (Table 2).

**TABLE 2:** Repair/regeneration effects of different cell types in the inner ear and exosome-based interventions.

Target cell types	Protective, reparative, or regeneration-related effects	Exosome source or engineering strategy and payload	Key molecules or pathways	Model system, species, and research type	Route of administration	Evidence level for exosome-based intervention
Hair cells	Inhibits apoptosis and reduces cisplatin-induced hair cell loss Functional auditory outcome: ABR/DPOAE not assessed	MSC-derived exosomes Payload: natural exosomes HSP70	HSP70-related pathways	*In vitro* auditory explant Model species: mouse cochlear explant Study type: *in vitro* study	Exosomes were directly added to/pre-incubated with the mouse cochlear explant culture system before exposure to cisplatin.	Direct cochlear exosome evidence (Park et al., 2021)
Hair cells	Inhibits oxidative stress and apoptosis, and reduces gentamicin-induced hair cell damage Functional auditory outcome: ABR/DPOAE not evaluated	Exosomes derived from mouse inner ear stem cells Payload: endogenous exosome miR-182-5p, targeting FOXO3	miR-182-5p/FOXO3 axis	*In vitro* model of hair cell damage Model species: mouse-derived HEI-OC1 auditory cell Study type: *in vitro* study	Directly add/co-incubate exosomes into the gentamicin-treated HEI-OC1 cell culture system.	Direct cochlear exosome evidence (Lai et al., 2020)
Hair cells	Lower the hearing threshold, reduce hair cell loss, and alleviate oxidative stress Functional auditory outcome: Reduced ABR thresholds at 8, 16, 24, and 32 kHz, and decreased hair cell loss	Exosomes derived from bone marrow MSCs Payload: natural/hypoxia-preconditioned MSCs exosomes and HIF-1α-related antioxidant effects	Regulation of oxidative stress	A mouse model of cisplatin-induced ototoxicity Model species: C57BL/6 mice Study type: *in vivo* animal study	Intratympanic administration	Direct cochlear exosome evidence (Yang et al., 2022a)
Hair cells	Reducing hearing loss caused by noise exposure Functional auditory outcome: detection of reduced ABR thresholds and improved DPOAE responses	Source and payload: exosomes derived from human MSCs	Not reported	Noise exposure model Model species: mouse Study type: *in vivo* animal study	Local intracochlear administration via the posterior semicircular canal.	Direct cochlear exosome evidence (Warnecke et al., 2020)
Hair cells	Reduce inflammatory factors and suppress caspase-3-related signaling Functional auditory outcome: reduced ABR threshold shift and hair cell apoptosis	Exosomes derived from spiral ganglion progenitor cells Payload: Natural exosomal miRNAs, including miR-21-5p, miR-26a-5p, and miR-181a-5p	IL-6, IL-1β, TNF-α, COX-2, and caspase-3	Ischemia-reperfusion model Model species: C57BL/6 mice Study type: *in vivo* animal study	Local cochlear administration	Direct cochlear exosome evidence (Yang et al., 2021)
Hair cells	Activate autophagy to reduce neomycin-induced hair cell loss Functional auditory outcome: reduced ABR thresholds at 4, 8, 16, 24, and 32 kHz	Source and payload: MSC-derived exosomes	Autophagy process	Neomycin-induced injury model Model species: HEI-OC1 cells, mouse cochlear explants, and C57BL/6 mice Study type: *in vitro*, *ex vivo*, and *in vivo* animal studies	Direct treatment *in vitro*/*ex vivo* culture, and round window niche injection for animal experiments.	Direct cochlear exosome evidence (Liu et al., 2024)
Spiral ganglion neurons and synapses	Promoting synaptic regeneration is a key target for neurotrophic support Functional auditory outcome: improved ribbon synapses, ABR wave I, or auditory threshold	Direct intervention by exosomes has not yet been established; engineered exosomes could be designed based on NT-3/BDNF Payload: NT-3 protein/gene/analogue or BDNF-PLGA gel	NT-3, BDNF	Excitotoxic injury, acoustic injury, models of age-related degeneration Model species: mouse cochlear cultures/explants and mice Study type: combined *in vitro*/*ex vivo* and *in vivo* animal studies	Treatment with culture system, NT-3 overexpression, round window NT-3, or middle ear BDNF-PLGA gel.	Indirect exosome evidence (Wang and Green, 2011; Wan et al., 2014; Suzuki et al., 2016; Kempfle et al., 2021; Cassinotti et al., 2022; Yu et al., 2024)
Spiral ganglion neurons and synapses	Regulate the local immune microenvironment and maintain synaptic stability Functional auditory outcome: synaptic or SGN survival	Exosomes can be engineered to carry Fractalkine signaling molecules Payload: CX3CL1, CX3CR1, AAV-NT-3	Fractalkine/ macrophage signaling	A model of spontaneous recovery from selective hair cell damage following noise exposure Model species: mice and guinea pigs Study type: *in vivo* animal study	Using gene/receptor animal models and AAV-mediated NT-3 overexpression in the cochlea.	Indirect exosome evidence (Kaur et al., 2015; Chen et al., 2018; Kaur et al., 2019)
Supporting cells (Lgr5 + and others)	Transformation into hair cell-like cells, enabling hair cell regeneration Functional auditory outcome: generation, maturation, markers, morphology, or partial functional characteristics of hair cell-like cells.	In the future, exosomes could be engineered to simultaneously deliver a combination of reprogramming factors Payload: Atoh1, Gfi1, Pou4f3, p27Kip1, GATA3, LIN28B/let-7, β-catenin-related regulatory factors	Atoh1, Gfi1, Pou4f3, p27Kip1, GATA3, LIN28B/let-7, β-catenin	Newborn and adult mice, acoustic injury model Model species: neonatal/postnatal and adult mice, cochlear explants, supporting cell cultures, and organoid/cell systems Study type: combined *in vitro*/*ex vivo* and *in vivo* animal studies	Using lineage tracing, genetic/viral manipulation, cochlear culture/organoid, or small molecule/siRNA reprogramming.	Theoretical engineering strategy (Bramhall et al., 2014; McLean et al., 2017; Kelly et al., 2012; Kuo et al., 2015; Walters et al., 2017; Li and Doetzlhofer, 2020; Chen et al., 2021; Tao et al., 2021; Iyer et al., 2022; Li et al., 2022; Nguyen et al., 2023; Quan et al., 2023)
Neurovascular units of the cochlea (endothelial cells, pericytes)	Improves microvascular permeability and promotes endothelial repair and angiogenesis Functional auditory outcome: no auditory functional outcome, assessing vascular/endothelial survival, permeability, migration, angiogenesis, and re-endothelialization	Exosomes derived from endothelial progenitor cells Payload: EPC exosome natural payload, miR-126/miR-126a-5p	miR-126a-5p	Models of ischemia and stroke in the brain and other tissues Model species/systems: mice, rats, and endothelial cell/BMEC culture models Study type: *in vitro* and *in vivo* animal studies	Systemic administration of EPC exosomes in animal models, and direct treatment in endothelial cell/BMEC cultures.	Indirect exosome evidence (Zhou et al., 2018; Wu et al., 2018; Li et al., 2016; Zhang et al., 2024)
Neurovascular unit of the cochlea (barrier structure)	Maintain the integrity of the blood-labyrinth barrier/blood-brain barrier and restore perfusion Functional auditory outcome: no auditory functional outcome was assessed. Evaluation focused on BBB/BSCB permeability, cerebral blood flow, vascular integrity, and neurological recovery.	Exosomes derived from neural progenitor cells and MSCs Payload: natural exosome payload	NF-κB, TIMP2/MMP, Caveolin-1/CD147/VEGFR2	Stroke and Spinal Cord Injury Models Model species: stroke mice, spinal cord injury rats, and endothelial/barrier cell models Study type: *in vitro* and *in vivo* animal studies	Systemic administration of exosomes in stroke/spinal cord injury animal models, with direct treatment in endothelial/barrier culture systems.	Indirect exosome evidence (Zhang et al., 2021; Barzegar et al., 2021; Xin et al., 2021)

Accordingly, vesicles derived from the inner ear are more likely to maintain signal preferences consistent with the condition of cochlear cells than exosomes from general sources. This functional advantage allows them to more precisely address the actual needs of the target tissue. Exosomes protect hair cells by regulating common downstream pathways, including oxidative stress, inflammatory cascade reactions, and programmed cell death (Park et al., 2021). Direct reprogramming of the hair cells themselves is not necessary for the survival benefit mechanism of hair cells. Research has indicated that addressing the local inflammatory microenvironment in the cochlea through exogenous intervention can effectively limit the release of pro-inflammatory cytokines, reduce excessive infiltration of immune cells, and subsequently restore tissue homeostasis.

Hair cells can evade ongoing inflammatory damage in this scenario of steady-state recovery, and the overall repair of the local microenvironment results in the maintenance of their function and structural integrity. The ability of hair cells to recognize and integrate vesicular signals is an important endogenous factor influencing their ultimate level of response to treatment. According to recent research, the primary roles of exosomes at the hair cell level include effectively reducing oxidative stress, alleviating inflammatory responses, and preventing programmed cell death (Liu et al., 2024).

However, the current evidence does not support the regeneration of high-quality, functional new hair cells within the cochlea of adult mammals. The practical use of engineered exosomes at the hair cell level is more appropriate as a tissue protector before regeneration and as an environmental regulator during the regeneration process. It is important to comprehend both the protective effect of hair cells and the limits of research design. Animal acute ototoxicity or noise models currently provide research data; however, human SNHL has a heterogeneous etiology, chronic degeneration, delayed treatment, and significant individual variability. It is also required to combine hair cell morphological protection, decreased oxidative stress, and enhanced ABR threshold, long-term threshold stability, and independent replication cohorts to demonstrate long-lasting efficacy.

### 4.2 Indirect exosome-mediated repair strategies of spiral ganglion neurons, supporting cells, and the cochlear neurovascular microenvironment

#### 4.2.1 Mechanisms of repair and remodeling in spiral ganglion neurons

At present, small exosomes high in BDNF in noise-induced hearing loss models provide direct evidence for neurotrophic protection, while research on NT-3-related synaptic repair serves as the theoretical foundation for cargo selection in future engineering exosome design. NT-3 encourages synaptic regeneration in inner hair cells and spiral ganglion neurons after excitotoxic injury, indicating that neurotrophic factors remain the primary biological foundation for the repair of spiral ganglion neurons (Wang and Green, 2011). Additionally, NT-3 can modulate synaptic density in the cochlear stria and stimulate synaptic regeneration after acoustic injury (Wan et al., 2014). The main goal of this approach is to accomplish a significant reconstruction of the structure and function of synaptic units, as indicated by an in-depth analysis of the effectiveness of NT-3 intervention and spiral ganglion repair. Local NT-3 administration through the round window stimulates synaptic regeneration in the cochlea after acoustic trauma (Suzuki et al., 2016). The adult cochlea maintains some degree of synaptic plasticity, further indicating that the structural reorganization and functional recovery of neural circuits have a clear physiological foundation, provided that the delivery location is sufficiently precise and the time window for action is sufficiently long.

Recent studies indicate that fibers with low spontaneous discharge rates are more susceptible to damage in noise-induced hidden hearing loss (Furman et al., 2013). The variations in the contributions of different nerve fiber subtypes to sound encoding must be considered while administering relevant treatments on spiral ganglion neurons. The repair of spiral ganglion neurons requires, at a minimum, sustained neurotrophic support and moderate, controlled neuroimmunomodulation, which enable the formation of neural connections and the maintenance of their stable existence by local tissues (Kaur et al., 2015; Chen et al., 2018). The recovery of spiral ganglion neurons appears to be a time-window-dependent process of synaptic reconstruction, as evidenced by the distinct temporal dynamics of the loss of ribbon synapses and related changes in postsynaptic structures after noise exposure (Liberman et al., 2015; Kaur et al., 2019). A small-molecule NT-3 analog can simultaneously promote axonal outgrowth and improve synapse formation *in vitro*.

NT-3 overexpression in the cochlea during middle age has been demonstrated to prevent age-related inner hair cell (IHC) synaptic dysfunction and slow hearing loss. Its use in cochlear repair encompasses both acute injury and chronic degenerative conditions, and goes beyond the benefits of post-injury treatment (Kempfle et al., 2021; Cassinotti et al., 2022). In terms of delivery strategy, a single controlled-release administration of a BDNF-containing hydrogel to the middle ear can markedly lessen IHC synaptic lesions and enhance hearing recovery (Yu et al., 2024). A crucial insight into the future direction of engineered exosomes is the fact that existing research offers a strong basis for defining the cargo selection, assessing efficacy, and identifying the optimal intervention windows for pathways, including NT-3, BDNF, chemokine regulation, and synaptic protection. The goal of using exosomes for spiral ganglion repair focuses on integrating the well-established neural repair mechanism into an engineered carrier that can achieve targeted delivery.

Human SNHL is linked to synaptic degeneration, neurotrophic deficiency, and inflammatory regulation. The potential for clinical translation in spiral ganglion and synaptic repair is indicated by the ability of BDNF/NT-3 to serve as engineered cargo for exosomes. However, much evidence currently available for synaptic repair comes from non-exosome delivery, gene overexpression, hydrogels, and *in vitro* models. These methods only indicate the mechanisms and intervention windows that can be further investigated; they cannot demonstrate that engineered exosomes have been able to stably restore synaptic structure and neural function in adults with SNHL. Future studies should include synaptic counting, ABR, DPOAE, neuronal survival, long-term follow-up, and independent replication cohorts.

#### 4.2.2 Supporting cell-mediated regenerative mechanisms

Supporting-cell plasticity offers a significant biological basis for future regeneration strategies based on exosomes, unlike the data directly mediated by exosomes in the cochlea. New hair cells in the postnatal cochlea originate from Lgr5-positive supporting cells. These cells produce high-purity sensory-like hair cells after clonal expansion. This suggests that supporting cells constitute a regenerative reserve that can be mobilized and offers direct cytological evidence that endogenous regeneration can be activated by delivering reprogramming factors (Bramhall et al., 2014; McLean et al., 2017). These studies did not establish exosome-mediated functional regeneration in the adult cochlea, but they did identify potential targets. Atoh1 contributes to the formation of the sensory epithelial mosaic in the mammalian cochlea and promotes cell proliferation-related regulatory events during the postnatal period. Inhibition of the Notch signaling pathway effectively induces hair cell regeneration after acoustic injury and results in corresponding restoration of hearing function; synergistic activation of signaling pathways such as Wnt/β-catenin on this basis can further improve the induction efficiency and survival of newly generated hair cells (Kelly et al., 2012; Kuo et al., 2015).

Collectively, these studies indicate the crucial significance of Atoh1-related pathways in regulating hair cell fate and their potential therapeutic value in regenerative medicine across three dimensions: developmental regulation, injury regeneration, and signaling synergy. *In vivo*, p27Kip1, GATA3, ATOH1, and POU4F3 work synergistically to promote the transdifferentiation of non-sensory cells into cochlear hair cells. The LIN28B/let-7 molecular axis can further control the capacity and effectiveness of neonatal mouse auditory supporting cells to differentiate into hair cells through the mTOR signaling pathway (Walters et al., 2017; Li and Doetzlhofer, 2020). The plasticity of supporting cells is jointly modulated by developmental transcription factors, cellular metabolic states, and growth conditions. However, this regenerative ability is markedly limited in the mature cochlea.

A single fate-determining factor is insufficient to effectively restore the regenerative capacity of supporting cells, and the low efficiency of cochlear regeneration in adults is attributed to a significant decline in the overall foundation of cellular plasticity as the organ matures. More mature hair cells form in the cochlea after birth due to the co-expression of Gfi1, Pou4f3, and Atoh1. It is challenging for a single transcription factor to independently complete the entire phenotypic remodeling process, and the stable establishment of a mature hair cell-like phenotype usually needs a regulatory mechanism that includes the coordinated activity of numerous factors (Chen et al., 2021). Furthermore, enhancer inactivation is a critical epigenetic barrier to the regeneration of sensory hair cells, whereas epigenetic changes and intercellular signaling jointly limit the reprogramming of ATOH1, GFI1, and POU4F3 in the cochlea of mature mammals (Tao et al., 2021; Iyer et al., 2022).

Current research suggests that sustaining cell regeneration is comparatively simple during the neonatal stage, but it is quickly impeded throughout the mature stage due to global silencing of gene regulatory regions. Follistatin increases the effectiveness of LIN28B-mediated reprogramming in these circumstances, although epigenetic modifications such as DNA methylation still silence hair cell-related gene networks. A combined strategy using small-molecule compounds and siRNA can help cellular reprogramming and generate hair cell-like cells in adult mice (Li et al., 2022; Nguyen et al., 2023; Quan et al., 2023). When it comes to clinical translation, mature phenotype, synaptic integration, electrophysiological function, long-term survival, and reproducible hearing recovery must be combined. However, current supporting cell research can help identify the regenerative factors that exosomes will deliver in the future.

#### 4.2.3 Neurovascular microenvironment remodeling

Exosome evidence from non-cochlear barrier systems suggests that neurovascular microenvironment remodeling is a promising approach for engineering exosome therapy for SNHL. BLB and related microvascular structures gradually undergo degenerative changes with age; these changes together provide a significant pathological foundation for lesions in the sensory epithelium of the cochlea (Neng et al., 2015). The structural and functional condition of the neurovascular microenvironment has a direct impact on the effectiveness of initiating the regenerative process and the subsequent capacity to sustain a stable phenotype, which may affect regeneration-related repair responses in regenerative therapy research. Exosomes derived from sources including endothelial progenitor cells and MSCs have been demonstrated to improve microvascular permeability, endothelial cell migration and proliferation, and vascular repair (Li et al., 2016; Wu et al., 2018; Zhou et al., 2018).

According to recent research, exosomes can be engineered as therapeutic tools that prioritize the stabilization of the vascular endothelium and the restoration of local perfusion. Exosomes derived from neural progenitor cells can improve blood-brain barrier integrity through NF-κB-mediated signaling, whereas exosomes derived from human placental MSCs can improve neurological outcomes following stroke by preserving cerebral blood flow (Barzegar et al., 2021; Zhang et al., 2021). These results imply that a key strategy to reestablish organ function is targeted microenvironment repair.

Particularly, stable maintenance of barrier structures and increased local perfusion jointly contribute to the overall reconstruction of the tissue microenvironment during microenvironment repair. Perivascular cells in the stria vascularis determine the stability of blood vessels and influence the survival of auditory neurons in the inner ear system (Zhang et al., 2023). The neurovascular microenvironment provides sensory cells with crucial metabolic support and plays a key regulatory role in maintaining the long-term homeostasis of neurons. These findings provide a theoretical basis for future exosome-based interventions in the cochlear neurovascular microenvironment.

For instance, exosomes maintain the integrity of the blood-brain barrier by inhibiting pathways involving caveolin-1, CD147, VEGFR2, and MMPs. Exosomes derived from EPCs deliver miR-126a-5p, thereby alleviating damage to cerebral microvascular endothelial cells and restoring cell viability, proliferation, and angiogenic capacity (Li et al., 2024; Zhang et al., 2024). Vascular homeostasis is a critical upstream factor regulating the long-term fate of outer hair cells, and restoring vascular endothelial signaling in the cochlea and alleviating vascular pathological alterations can notably halt the progressive death of outer hair cells and the steady decline in hearing (Patel et al., 2024).

These studies indicate that the neurovascular microenvironment acts as the fundamental tissue basis for maintaining the long-term stability of associated structures and functions (Xin et al., 2021). This aspect has not received enough attention in previous studies, which has made it challenging for many effective interventions that target sensory cells to eventually achieve long-lasting improvements in auditory function. It is important to demonstrate cochlear distribution, perfusion improvement, ABR, DPOAE, and long-term safety in the SNHL model. The non-cochlear neurovascular microenvironment protection results can be used as evidence for hypothesis generation.

## 5 Challenges and prospects for engineered exosomes in SNHL treatment

### 5.1 Key technical challenges in the engineering of exosomes for SNHL treatment

The main obstacle to the effective application of engineered exosomes in clinical settings is the seamless integration of the raw material system, critical quality attributes, release criteria, and scalable manufacturing processes. This integration must produce a comprehensive development cycle that can support both Good Laboratory Practice evaluation and Good Manufacturing Practice (GMP) clinical production (Wang et al., 2024; Humbert et al., 2025).

Exosomes are regarded as a next-generation delivery platform. Their excellent natural biocompatibility, complex membrane characteristics, and capacity to cross biological barriers make them appealing (Murphy et al., 2019; Herrmann et al., 2021). These features make exosomes markedly more complex than conventional nanocarriers in terms of composition definition, homing behavior and functional consistency. Even though bioactive vesicle samples can be obtained for most current studies, there remains a gap between these efforts and the large-scale production of preparations with uniform quality, stability, clear quality standards, and repeatable biological effects.

The current development paradigm is shifting from proof-of-concept studies toward scalable and continuous manufacturing processes. Key quality aspects of the final product can be altered by even minor differences in donor cell sources, culture conditions, separation and purification schemes, or engineering transformation (Claridge et al., 2021; Estes et al., 2022). It is recommended to provide data, including particle counting, protein quantification, biomarker analysis, single-particle characterization, contaminant control, and functional assays, to establish a truly comparable evidence basis across various studies (Welsh et al., 2024). This is the fundamental prerequisite for transforming exosome treatment from experimental materials to standardized products. Different loading methods for drug-loaded exosomes simultaneously change the amount of residual free drug, the distribution of particle sizes, and the integrity of the vesicle membrane, all of which have an impact on the final functional output (Koehler et al., 2017; Wang et al., 2025).

Research on therapeutic exosomes for SNHL should report at least the following to aid in the implementation of the previously mentioned recommendations: (1) donor cells identification and source; (2) culture conditions, such as culture media and additives, passage number, cell confluence, and any pretreatment; (3) exosome isolation and purification techniques; (4) particle concentration and size, along with the identification of markers related to exosomes and non-exosome components; (5) purity and contaminant testing, including residual proteins or lipoproteins, endotoxins, sterility, and, where applicable, mycoplasma; (6) loading efficiency and loading capacity of therapeutic payloads; (7) techniques for removing and quantifying unencapsulated or free payloads; (8) the particle-to-protein ratio as a method-dependent auxiliary indicator of purity; (9) efficacy testing related to the proposed mechanism of action and pertinent to the indications, such as inhibiting oxidative stress or apoptosis, protecting cochlear cells, or assessing neurotrophic activity; (10) formulation and storage conditions, such as temperature, duration, and freeze-thaw history; (11) predetermined release criteria, as well as reproducibility between independently prepared batches. To link biological activity to product quality and facilitate efficient comparisons between studies, the previously mentioned product quality data should also be reported for SNHL applications along with dosage units, administration routes, cochlear exposure or biodistribution, and auditory outcomes.

For instance, the dose window that the cochlea can tolerate is comparatively narrow in SNHL. A short-term protective effect may be observed if the carrier, free drug, and release profile are not clearly distinguished. However, it is challenging to ascertain whether the actual effect is caused by the effective delivery of drugs by the exosomes or by the simultaneous existence of free drugs. Exosomes are more similar to the physiological environment than well-established nanocarriers such as liposomes.

Therefore, they pose greater challenges for pharmacokinetic characterization, surface functional consistency, and batch repeatability (van der Koog et al., 2022). The natural origin of exosomes will not always make them convenient for development, and each engineering step will probably have an impact on their overall properties. Storage and transportation are more than merely post-processing steps. Temperature regulation, lyoprotectant systems, and reconstitution conditions can directly impact the particle size distribution, structural integrity, and biological activity of exosomes.

Accordingly, the stability of the preparation is an unavoidable engineering variable in clinical development (Dogan et al., 2025). Preclinical data lack comparability. This is mostly caused by variations in preparation, post-treatment, and storage conditions, which directly result in notable alterations in the physicochemical and biological properties of vesicle preparations, in addition to the inherent biological differences. An assessment framework that can consistently connect loading behavior, quality features, cochlear exposure, and functional results is necessary for developing exosome-based therapy for SNHL.

From a regulatory perspective, exosomes combine characteristics of biological products, complex mixtures, and delivery vehicles, resulting in the absence of standardized procedures for raw material management, release standards, efficacy testing, and long-term safety assessment (Wang et al., 2024). A systematic development framework that can establish a consistent correlation between process parameters, quality attributes, *in vivo* distribution, and clinical endpoints is currently required from a translational perspective (Ghodasara et al., 2023). Strong translational value can only be achieved when a positive result simultaneously satisfies the following criteria: A clinically relevant model, precise characterization of exosome preparation, a defined dosage and administration route, confirmed cochlear exposure, distinct functional hearing endpoints, and reproducible long-term efficacy. Nowadays, many studies merely meet some of these requirements. Although the protective effect exhibited in rodents suggests the exosome intervention is feasible, the certainty of translation to human SNHL is diminished by insufficient reproducibility, inadequate standardization of exosomes, unclear dosage equivalence, limited stratification by gender and age, and short follow-up periods. There are notable variations in product sources, administration routes, dosage units, and endpoints in early human research.

Products used for perioperative intra-abdominal administration, intravenous administration, and local skin injection are defined and evaluated differently (Nassar et al., 2016; Warnecke et al., 2021; Johnson et al., 2023). Engineered exosomes still have a long way to go before they are developed into actual otological clinical products in SNHL due to issues such as the limited treatment window for cochlear drug delivery, the difficulty of obtaining local tissue samples, and the length of time needed to achieve long-term auditory effects. However, the etiology of SNHL varies greatly. There are difficulties, particularly with regard to therapy timing, primary target cells, and reversibility, because acute noise-induced hearing loss, cisplatin-induced ototoxicity, ischemia-reperfusion injury, age-related degeneration, and hereditary hearing loss differ from one another. To develop products with precise indications and a perfect clinical pathway, these intrinsic differences must be addressed.

### 5.2 Clinical translation prospects and future research directions for engineered exosomes in SNHL treatment

To ensure the stability and repeatability of the treatment effect, the focus of research on engineered exosomes has shifted from verifying efficacy to optimizing the administration route for particular indications and establishing a quality control system. To provide a more comprehensive framework for product development, early translational research in the field of exosomes has started to expand beyond basic safety studies and include GMP-compliant production, quality control, and procedures consistent with clinical trials (Humbert et al., 2025). In terms of the inner ear, the first clinical application of exosomes in the human cochlear scala tympani demonstrates that the local cochlear administration is feasible, and preliminary results indicate that it is safe throughout the perioperative period. Although its clinical efficacy has not yet been proved for SNHL, this research serves as a foundation for subsequent early clinical trials of SNHL for certain indications (Warnecke et al., 2021). Prospective controlled studies are necessary to assess the following important issues: local and systemic safety, appropriate dosage and dosing interval, repeated treatment, immunological response, cochlear and systemic biodistribution, and long-term auditory outcomes. Currently, the primary value of this research is that it offers initial proof of viability.

To address this and lessen the need for trial-and-error in engineering design, it is an important future research direction to use machine learning and artificial intelligence to develop multivariate optimization models that incorporate elements such as ligand screening, drug combinations, formulation parameters, and *in vivo* distribution (Greenberg et al., 2023). Since exosome engineering is a system with several connected variables, optimizing one of them usually only improves local indicators and does not always result in an overall increase in therapeutic efficacy. Development efficiency may be hampered by the complexity of the exosome platform. This requires an improved evaluation model.

For instance, it is possible to assess the functional impacts of hair cell differentiation, cytotoxicity, and potential therapeutic approaches in a system that more closely resembles human developmental processes in the establishment of human-derived stem cell-based inner ear organoids (Haraszti et al., 2018). The quality of sensory epithelial lineage reconstruction is enhanced by a new generation of high-fidelity cochlear organoids, which offer a model foundation for screening engineered exosomes to find receptor targets and therapeutic windows that more closely mimic those of the human cochlea (Moore et al., 2023).

Organoids can closely replicate the cellular composition of the human cochlea and vestibular organs at a higher resolution, which helps establish a more reliable efficacy evaluation platform, according to a study comparing human inner ear organoids with primary tissue at the single-cell level (van der Valk et al., 2023). These models are useful because they can start combining the assessment of cochlear toxicity, cell type-specific response, and targeting into a single experimental framework. This allows for a gradual transition from the previous evaluation method, which primarily relied on animal-based endpoint tests, to mechanism-based assessments that are more similar to the condition of human tissues. Different cell groups in the cochlea exhibit distinct transcriptional characteristics linked to stress, inflammation, and repair following noise-induced damage. This offers a new direction for the design of engineered exosome cargoes, ranging from intervention for certain cell states to broad-spectrum protection (Milon et al., 2021).

Consequently, the single-cell atlas of the mouse cochlea is starting to provide a molecular roadmap for cell-specific targeted delivery, allowing for the more precise design of promoters and the selection of surface ligands at the cell-type level (Jean et al., 2023). Different populations of cochlear cells are susceptible to issues at different phases of the aging process, according to a single-cell atlas of the aging cochlea. Exosomes that target age-related SNHL have a different design rationale than those that target acute ototoxicity or noise-induced hearing loss (Sun et al., 2023).

According to current research, the main objectives of future delivery strategies are to precisely target particular populations of cochlear cells within the cochlea, optimize the duration of action, and guarantee alignment with the pathological condition. The capacity of the platform to precisely match various etiologies, disease courses, and cellular states with certain cargo combinations and surface designs makes it competitive. The future advantage of engineered exosomes lies in their potential to lead the shift from tissue-level delivery to cell-level delivery.

## 6 Conclusion

Engineered exosomes are creating novel therapy options for SNHL by moving the focus from functional compensation to etiological intervention and targeted repair. The domains of exosome engineering covered in this review include source selection, pretreatment, surface targeting, therapeutic cargo loading, and delivery route optimization, all of which combined constitute a systematic strategy. To highlight the interdependence between targeted delivery and microenvironmental remodeling in the treatment of SNHL, this review summarizes the available data in the three areas of exosome engineering, BLB crossing, and cochlear protection, repair, and regeneration-related techniques.

Engineered exosomes are anticipated to have a wide range of effects in hair cell protection, spiral ganglion neuron protection, supporting-cell plasticity, and neurovascular microenvironment remodeling in addition to improving the local accumulation of therapeutic molecules in the cochlea after crossing the BLB by regulating inflammatory responses, oxidative stress, apoptosis, neuroprotection, and microenvironmental homeostasis. Engineered exosomes are evolving from simple delivery carriers into multipurpose therapeutic platforms that integrate transport and regulatory roles. Current research supports the potential of engineered exosomes for targeted drug delivery and cochlear microenvironment remodeling. Applications related to regeneration are still in the research stage and are primarily supported by indirect or preclinical evidence. However, important obstacles such as effectively crossing the BLB and optimizing targeted modification must be solved for exosomes to truly transition from laboratory research to clinical application. These issues require interdisciplinary cooperation and coordination in the domains of materials science, nanotechnology, and clinical medicine. Systemic challenges in the clinical translation of exosomes include the establishment of a standardized and scalable production system, developing personalized and stratified treatment approaches, and a comprehensive evaluation of the long-term safety and immunological impact *in vivo*. A closed-loop system integrating innovation and interdisciplinary collaboration must be established across disciplines, including basic mechanisms, engineering and technologies, and clinical evaluation. However, it has not yet been proven that the cochlea of adult mammals robustly generates mature, synaptically integrated, and functional new hair cells.

Therefore, engineered exosomes should currently be considered primarily as protective and delivery platforms. Their application in regeneration-related strategies is still investigational. Standardized exosome characterization, clinically relevant models, reproducible hearing results, and long-term safety verification are necessary when pursuing clinical translation. It is anticipated that this would enable clinical translation in the field of SNHL, offering patients with various causes and subtypes of hearing impairment more accurate and efficient treatment options.
